# Improved Quality Control Method for Prescriptions of *Polygonum capitatum* through Simultaneous Determination of Nine Major Constituents by HPLC Coupled with Triple Quadruple Mass Spectrometry 

**DOI:** 10.3390/molecules181011824

**Published:** 2013-09-25

**Authors:** Kai-Xia Zhang, Yue-Sheng Wang, Wen-Guang Jing, Jun Zhang, An Liu

**Affiliations:** 1 Institute of Chinese Materia Medica, China Academy of Chinese Medical Sciences, Beijing 100700, China; 2 Jiangxi University of Traditional Chinese Medicine, Nanchang 330006, China; 3 Hunan University of Chinese Medicine, Changsha 410208, China

**Keywords:** *Polygonum capitatum*, Relinqing^®^, HPLC-QQQ MS, quality control

## Abstract

As a traditional Miao-nationality medicinal plant, *Polygonum capitatum* has been used in clinical practice for several thousand years. Its prescriptions, including three dosage forms: granules, capsule and tablet are known by the brand name Relinqing^®^ and have played an indispensable role in the treatment of urinary system infection, pyelonephritis and kidney stones. However, no study about the comprehensive quality evaluation of Relinqing^®^ has been reported. In the present paper, a method for the simultaneous determination of nine major compounds in three dosage forms of Relinqing^®^ using HPLC coupled with triple quadrupole mass spectrometry (HPLC-QQQ MS) was established to comprehensively evaluate their quality. The nine compounds, including four phenolic acids, four flavonoids and a lignin, were analyzed with acceptable linear regression relationship (r^2^, 0.9923–0.9992), precision (RSD, 1.25%–2.78%), repeatability (RSD, 2.05%%–3.47%), stability (RSD, 1.84%–3.72%) and recovery (93.60%–108.54%, RSD ≤ 3.67%). The present study fills the gap in the multivariate quality control of Relinqing^®^ and provides a valuable reference for quality standards and dosage reforming of this traditional Chinese medicine.

## 1. Introduction

Traditional Chinese Medicine (TCM) has been used in clinical practice for several thousand years. TCM has played an indispensable role in the prevention and treatment of diseases, especially the complicated and chronic ones. In recent years, TCM has been attracting more and more attention because of its complementary therapeutic effects to Western medicines, and its capability to deal with many essential problems that have not been solved by conventional medicinal practices. However, the extreme complexity and uncontrolled quality of TCM preparations become the most important problems for the application and development of TCM. Conventional quality control mode is the simply quantitative analysis of one or only a few components, which cannot represent the real quality of herb medicine [1,2,3]. Quantitative analysis of multiple active components is becoming the most direct and indispensable method to move forward the development of TCM [4,5,6].

Relinqing^®^ which has been approved by the Chinese Food and Drug Administration, is commonly used with considerable therapeutic efficacy in the treatment of urinary tract infection, pyelonephritis, cystitis urinary calculi and other diseases [7,8,9]. The Relinqing^®^ capsule and tablets based on Relinqing^®^ granules are prepared proportionally from the TCM *Polygonum capitatum* Buch-Ham. ex D. As the main active ingredient of Relinqing^®^ and a traditional Miao-nationality medicinal plant, *Polygonum capitatum* is widely distributed in the minority districts in southwest China. Previous pharmacological studies have demonstrated that aqueous or ethanolic extracts of *P. capitatum* possessed antibacterial, analgesic, anti-inflammatory, hypothermia, diuretic, and anti-oxidative activities [10,11,12], while chemical investigations showed the presence of flavonoids, phenolic acids, lignans, triterpenoids, steroids, as well as other fatty acid esters in this plants [13,14,15].

Therefore, the active constituents of Relinqing^®^ include flavonoids, phenolic acids and schizandriside. Flavonoids, including catechin, quercetin, quercetin and kampferol are the main biologically active constituents in *P. capitatum* [16,17,18], which to some extent might explain the mechanisms of the clinical effects of this drug. Phenolic acids are simple non-flavonoid compounds constituting a large group of phenolic compounds which play an important role in the antibacterial activity and anti-inflammatory activity of Relinqing^®^. Besides, abundant and bioactive schizandriside has been found in Relinqing^®^ [19]. Therefore, flavonoids, phenolic acids and schizandriside contents can be an important index in the quality evaluation of Relinqing^®^ on account of their various pharmacological activities. Unfortunately, few studies on the quantitative determination of chemical constituents in Relinqing^®^ have been reported so far. In previous papers, some flavonoids and phenolic acids were determined by HPLC [20,21,22], but the results could not reflect the drug’s quality exactly and rapidly. In addition, these methods suffered from low resolution and sensitivity. Along with the development of analysis technology, triple quadrupole mass spectrometry (QQQ MS) provides a sensitive and accurate quantification method of multiple low-concentration compounds in the complex system by multiple-reaction monitoring (MRM) mode [23,24]. A rapid resolution liquid chromatography-QQQ MS method was much more suitable for the determination of components in Relinqing^®^ with short analysis time, wide linearity range and low LOD and LOQ.

In present study, nine major compounds including: (1) gallic acid; (2) protocatechuic acid; (3) vanillic acid; (4) syringic acid; (5) catechin; (6) schizandriside; (7) quercitrin; (8) quercetin and (9) kaempferol in 12 batches of Relinqing^®^ from three dosage forms were analyzed by HPLC-QQQ MS. A sensitive and accurate quantification method was established by multiple-reaction monitor (MRM) mode. Linear regression relationship (r^2^, 0.9923–0.9992), precision (RSD, 1.25%–2.78%), repeatability (RSD, 2.05%–3.47%), stability (RSD, 1.84%–3.72%) and recovery (93.60%–108.54%, RSD ≤ 3.67%), were presented. To the best of our knowledge, this is the first time that HPLC-QQQ MS was used to the simultaneous determination of flavonols, phenolic acids and lignans in Relinqing^®^. The results have indicated that this advanced method is fast, sensitive and convenient to show the real quality of the formula, which should benefit the quality control and clinical usage of Relinqing^®^.

## 2. Results and Discussion

### 2.1. Optimization of LC-MS/MS Conditions

**Figure 1:**
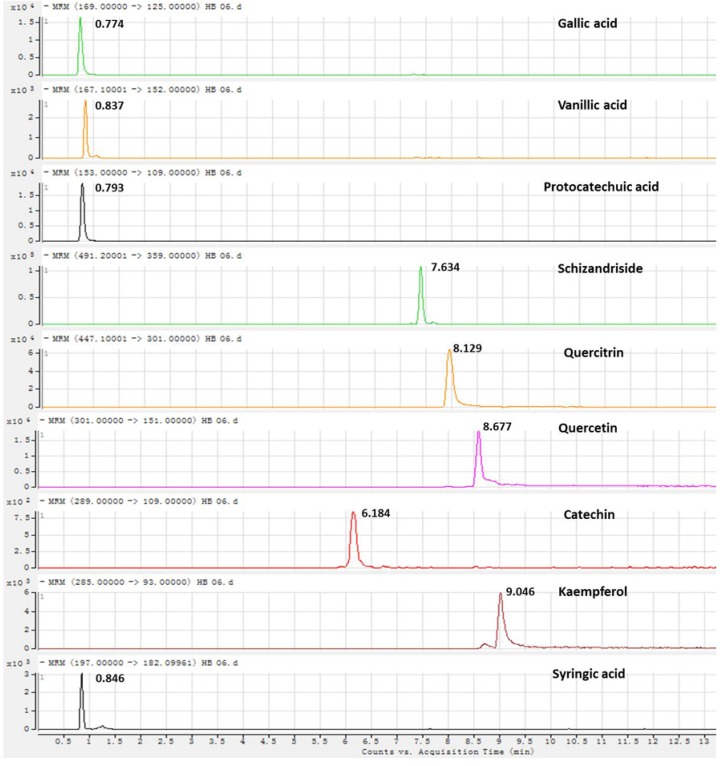
The RRLC-MS/MS analysis MRM chromatograms of the 9 markers.

Mobile phases such as methanol-water, acetonitrile-water, and aqueous acetonitrile-acid solvents were examined to achieve a suitable chromatographic behavior and a satisfactory MS response. The acetonitrile-water solvent provided better chromatographic resolution and MS ionization. Both positive and negative ionization modes were investigated. In the negative mode, the quasi-molecular and production ions were stable and reproducible [25], therefore, the negative ionization mode was chosen for subsequent experiments. In order to get more stable product ions and higher responses, Frag (fragmentor) and CE (collision energy) were optimized because of their important roles in ionization. The retention time (RT), MS information of the quantitative analytes and the HPLC-MS/MS chromatographic spectra of nine markers are listed in Table 1. The HPLC-MS/MS chromatographic spectra of the nine markers are shown in Figure 1.

### 2.2. Method Validation

#### 2.2.1. Linearity and Detection Limit

The calibration curves, plotted with a series of concentrations of standard solutions, were constructed from the peak-area ratios of each standard to internal standard *versus* concentrations of each analyte. Nine marker curves were all made at nine levels. Acceptable linear correlation and high sensitivity at these conditions were confirmed by correlation coefficients (r^2^, 0.9937–0.9992). The limits of detection (LODs) and quantification (LOQs) were calculated as 3- and 10-fold of the ratio of signal-to-noise (S/N); they were 1.0–5.0 ng·mL^−1^ and 5.0–10 ng·mL^−1^ for (1) gallic acid; (2) protocatechuic acid; (3) vanillic acid; (4) syringic acid; (5) catechin; (6) schizandriside; (7) quercitrin; (8) quercetin; (9) kaempferol. Detailed information regarding calibration curves, linear ranges, LODs and LOQs is summarized in Table 2.

**Table 1:** Characterization of 11 markers on the LC-MS and the optimized MRM parameters for quantification.

Compounds	Characterization of 11 markers			MRM parameters		
	RT (min)	M-H/M (*m/z*)	Lost ions	Quantification transition (*m/z*)	Frag (V)	CE (V)
Gallic acid	0.774	169	125[M-COOH]-; 79[M-COOH-3OH+3H]-	169→125	80	10
Protocatechuic acid	0.793	153	109[M-COOH-H]-; 91[M-COOH-H20]-	153→109	80	10
Vanillic acid	0.837	167	152[M-CH3]-; 123[M-COOH]-	167→152	80	10
Syringic acid	0.846	197	182[M-CH3]-; 153[M-COOH]-	197→182	80	20
Catechin	6.184	289	245[M-CH3CHO]-; 109[M-2OH-C6H5-CH3CHO-CH3]-	289→109	150	20
Schizandriside	7.634	491	359[M-xylose]-; 344[M-xylose-CH3]-	491→359	200	20
Quercitrin	8.129	447	301[M-rhamnose]-; 255[M-rhamnose-COOH]-	447→301	180	20
Quercetin	8.677	301	179[M-2OH-C6H4-CH2]-; 151[M-2OH-C6H4-CH3CHO]	301→151	150	15
Kaempferol	9.046	285	117[M-2OH-C6H6-CH2O-2H2O]-; 93[C6H5+OH]	285→ 93	150	25
Internal standard1	3.021	151	107[M-COOH]-; 92[M-COOH-CH3]-	151→107	80	5
Internal standard2	7.749	579	459[M-glucose+CH3COOH]-; 271[M-glucose-rhamnose]-	579→271	200	30

**Table 2:** Calibration curves, linear ranges, LOD and LOQ of the 9 markers.

Compounds	Calibration curves	Linear ranges (ng·mL^−1^)	Correlation coefficient	LOD (ng·mL^−1^)	LOQ (ng·mL^−1^)
Gallic acid	Y = 0.7624X − 1.8747	100–10,000	0.9937	5.0	10
Protocatechuic acid	Y = 0.9881X − 0.0169	5–500	0.9974	1.0	5.0
Vanillic acid	Y = 0.6889X − 0.6085	100–10,000	0.9982	1.0	5.0
Syringic acid	Y = 11.5119X + 68.0960	5–1,000	0.9992	1.0	5.0
Catechin	Y = 72.2795X + 637.9497	5–1,000	0.9965	1.0	5.0
Schizandriside	Y = 0.0019X − 0.0032	50–10,000	0.9971	5.0	10
Quercitrin	Y = 9.6795X − 0.8127	5–10,000	0.9989	1.0	5.0
Quercetin	Y = 0.7283X − 0.0865	50–10,000	0.9991	1.0	5.0
Kaempferol	Y = 0.3322X − 0.0300	10–5,000	0.9987	1.0	5.0

#### 2.2.2. Precision, Repeatability and Stability

The intra- and inter-day precisions were investigated to evaluate the precision of this method by measuring a mix standard solution consisting of eleven markers under the optimized conditions. Their relative standard deviations (RSD) were less than 2.36% and 2.78%, respectively. Six independent samples of Relinqing^®^ (G1, was randomly selected) were analyzed in parallel by the above-established method for the evaluation of repeatability. The RSD of nine standards were within the range of 2.05%–3.47%. The storage stability (RSD, n = 6) of the measurements for sample (G1, C1 and T1) solution was 1.84%–3.72% within 24 h in the room temperature. These results are displayed in Table 3.

#### 2.2.3. Recovery

Known amounts of each standard solution at high, middle and low concentration levels were mixed with known amounts of Relinqing^®^ samples (G1, C1 and T1). Then the samples were extracted and analyzed by the above-established method, and triplicate experiments were repeated at each level. As shown in Table 4, the recovery rate of nine standards varied from 93.60%–108.54% (RSD ≤ 3.67%), revealing the acceptable recovery and accuracy of this method.

**Table 3:** Precision, repeatability and stability of the 9 markers.

Compounds	Precision (RSD, n = 6) %		Repeatability (RSD, n = 6) %	Stability (RSD, n = 6) %		
	Intra-day	Inter-day		G1 C1 T1		
Gallic acid	1.68	2.11	2.75	2.27	2.35	3.16
Protocatechuic acid	1.59	2.35	2.17	2.38	2.67	2.78
Vanillic acid	2.36	2.47	3.03	2.79	2.08	2.91
Syringic acid	2.07	2.58	2.69	2.67	3.24	3.56
Catechin	1.73	2.72	2.78	3.29	2.49	2.44
Schizandriside	2.32	2.78	3.47	2.88	2.37	3.72
Quercitrin	1.25	1.47	2.05	1.84	2.16	2.38
Quercetin	1.54	1.87	2.19	2.17	3.24	2.27
Kaempferol	1.68	2.31	2.57	2.74	2.35	3.04

**Table 4:** Recoveries of the 9 markers (n = 3) in Relinqing^®^ granules, capsule and tablet.

Compounds	Granules		Capsule		Tablet	
	Recovery (%)	RSD (%)	Recovery (%)	RSD (%)	Recovery (%)	RSD (%)
Gallic acid	98.61%	2.18	99.50%	3.03	101.38%	2.13
	100.65%	2.33	96.91%	2.45	99.76%	2.65
	95.68%	2.46	101.05%	2.19	101.33%	2.29
Protocatechuic acid	97.34%	2.98	96.57%	3.17	96.57%	3.15
	98.29%	3.10	101.47%	2.27	102.25%	3.27
	102.51%	2.78	95.00%	3.66	97.70%	3.26
Vanillic acid	98.91%	2.59	104.50%	2.49	101.38%	3.55
	101.70%	2.84	97.70%	2.85	98.05%	2.64
	103.12%	3.14	108.54%	3.14	103.82%	2.74
Syringic acid	102.07%	3.19	102.72%	2.19	101.84%	2.15
	102.43%	3.28	98.13%	3.28	97.40%	3.28
	95.58%	2.97	95.29%	2.87	95.14%	2.97
Catechin	98.68%	2.46	103.88%	3.26	101.85%	2.46
	107.80%	2.79	95.79%	2.89	102.40%	2.79
	98.23%	3.13	95.93%	3.13	97.63%	3.03
Schizandriside	101.36%	3.28	101.98%	3.08	101.20%	3.48
	101.53%	3.24	95.01%	3.24	100.57%	2.14
	98.30%	2.85	97.94%	2.85	97.59%	2.15
Quercitrin	100.97%	3.77	101.22%	3.27	101.83%	3.67
	102.73%	3.54	101.53%	3.54	104.04%	3.34
	99.53%	3.42	98.27%	3.62	98.27%	2.62
Quercetin	97.13%	2.79	95.26%	2.79	101.89%	2.79
	104.62%	2.57	95.81%	2.57	97.74%	2.57
	97.62%	2.84	104.03%	2.84	96.30%	2.53
Kaempferol	93.60%	2.13	95.25%	2.23	97.15%	2.13
	105.60%	2.15	104.64%	2.15	102.32%	2.42
	95.07%	3.08	96.03%	2.38	97.43%	3.18

### 2.3. Sample Determination

The developed analytical method has been applied for the identification and quantification of nine target compounds in 12 batches of Relinqing^®^ samples. The contents of nine compounds, based on their respective calibration curves, were summarized in Table 5.

**Table 5:** Contents of the 9 markers in different Relinqing^®^ samples (ng·mL^−1^).

Samples	Contents of each compound in different relinqing samples								
	1	2	3	4	5	6	7	8	9
G1	21,751.04	568.31	463.72	68.92	56.31	852.04	699.64	84.32	46.95
G2	19,667.32	587.64	457.58	70.25	58.67	657.38	687.81	80.77	45.57
G3	20,996.64	495.94	471.46	69.67	53.71	789.68	696.79	93.94	44.82
G4	19,589.24	488.73	482.88	75.39	59.86	856.41	663.84	95.63	46,78
	20,501.06	535.15	468.91	71.05	57.14	788.87	687.02	88.66	45.78
RSD	5.14%	9.37%	2.32%	4.13%	4.76%	11.76%	2.36%	8.17%	2.36%
C1	17,855.36	359.67	134.37	58.98	138.68	679.78	609.64	185.37	47.81
C2	17,015.81	468,95	147.63	72.74	156.42	516.89	626.79	168,98	46.03
C3	19,389.43	443.64	159.83	65.81	140.17	591.57	614.46	174.46	44.42
C4	18,544.16	369,97	138.52	73.57	163.59	714,62	621.69	151.63	47.83
C	18,201.19	401.65	145.08	67.77	149.71	596.08	618.14	170.48	46.52
RSD	5.54%	14.78%	7.76%	10.05%	8.18%	13.67%	1.23%	10.09%	3.51%
T1	9,439.76	277.79	426.14	59.42	51.37	839.17	403.48	220.17	48.96
T2	12,920.56	321.46	437.27	66.89	59.47	765.04	409.79	160.79	42.37
T3	15,145.27	258.42	419.49	72.47	62.89	323.66	302.42	345.53	54.89
T4	11,075.44	306.54	403.72	58.81	60.34	691.74	423.54	193.41	46.54
T	12,145.26	291.05	421.65	64.39	58.51	654.9	564.50	229.97	48.19
RSD	20.21%	9.72%	3.32%	10.11%	8.51%	34.94%	14.43%	35.12%	10.85%
total	16,919.17	409.28	345.21	67.73	88.45	679.95	395.07	163.03	46.83

### 2.4. Discussion

In this paper, the HPLC method coupled with triple quadrupole mass spectrometry was proven to be a reliable and powerful technique for simultaneous determination of nine active components including four phenolic acids, four flavonoids and a lignan in three dosage forms of Relinqing^®^. The MRM mode of QQQ MS enabled identification of target compounds with high selectivity, even at very low concentrations by comparison with standards. In consideration of the different minor ingredients in different dosage forms of Relinqing^®^ which could cause the diversity of storage stability and recovery, storage stability and recovery were detected in three dosage forms respectively during the methodological study. Owing to the significant structural differences between flavonoids and phenolic acids, two internal standards were used to validate these two kinds of compounds, in order to improve the accuracy and rationality of the experiment. The samples of gallic acids were diluted 10-fold for their high content to make detection value satisfy the linear range.

**Figure 2:**
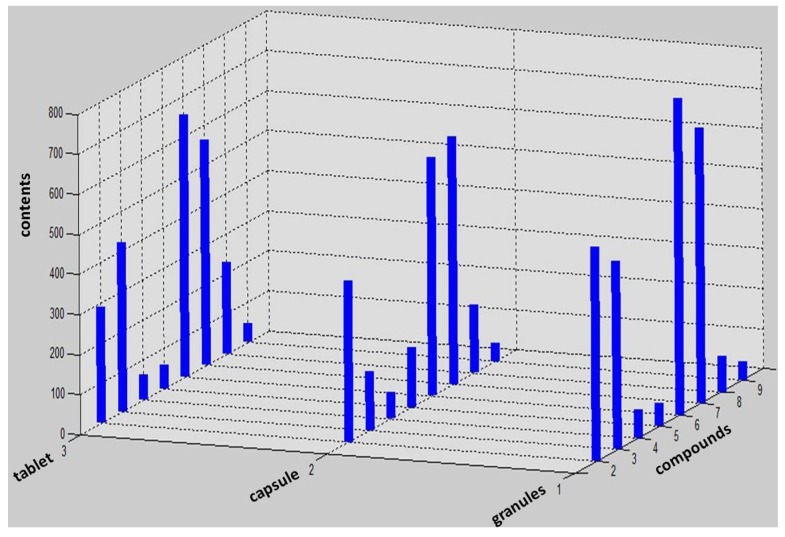
The average content of target markers except gallic acid in three dosage forms.

**Figure 3:**
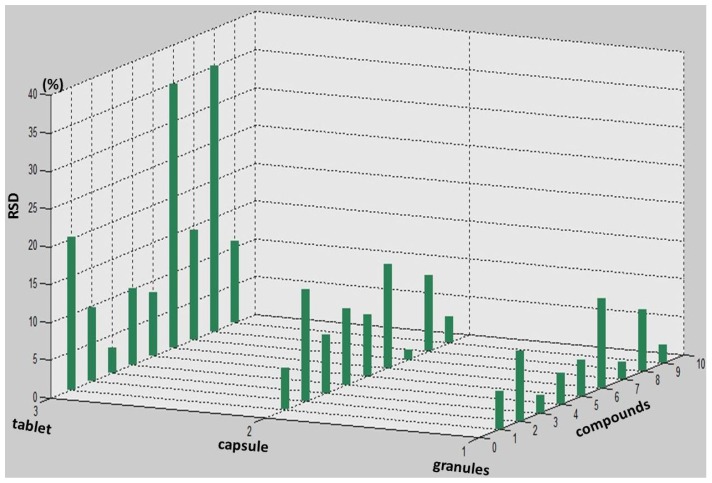
The RSD of the 9 markers in different batches of three dosage forms.

The gallic acid content should not be less than 23 mg in per Relinqing^®^ particles according to the Chinese Pharmacopeia (2010 Edition). Our experimental results showed that all the three dosage forms of Relinqing^®^ meet the national standard when measured under same dosage conditions. Apart from (1) gallic acid; (2) the contents of protocatechuic acid; (3) vanillic acid; (4) syringic acid; (5) catechin; (6) schizandriside; (7) quercitrin; (8) quercetin; (9) kaempferol, were analysed by MATLAB.According to the average content of target markers in three dosage forms (Figure 2) we suggest it’s more reasonable to use contents of (1) gallic acid; (2) protocatechuic acid; (6) schizandriside and (7) quercitrin together to control the quality of Relinqing^®^ because of their higher and stable amounts in different dosage forms. According to the comparison between different batches in the same dosage form (Figure 3), granules showed better stability than capsules and tablets, a forceful reference to optimizing the dosage of Relinqing^®^.

## 3. Experimental

### 3.1. Materials and Reagents

HPLC grade methanol (Fisher Scientific, Waltham, MA, USA), acetonitrile (Fisher Scientific) and deionized water obtained from a Milli-Q water system (Millipore Corp., Bedford, MA, USA) were used for preparation of mobile phases. All solvents were degassed by ultrasound and an on-line degassing system.

Twelve batches of Relinqing^®^ including four batches of granules G1-G4 (batch No.: from 120202 to 120406), four batches of capsules C1-C4 (batch No.: from 20120710 to 20121102), and four batches of tableta T1-T4 (batch No.: from 20120514 to 20120801) were respectively collected from Guizhou province and Beijing in China.

Standards of: (1) gallic acid; (2) protocatechuic acid; (3) vanillic acid; (4) syringic acid; (5) catechin; (6) schizandriside; (7) quercitrin; (8) quercetin and (9) kaempferol were isolated from *Polygonum capitatum* and identified by our laboratory. *p*-Methoxybenzoic acid (internal standard 1) and naringin (internal standard 2) were purchased from Beijing Bailingwei Technology Ltd. (Beijing, China). The purity of all compounds was more than 98% (as determined by HPLC).

### 3.2. Preparation of Sample Solutions

Twelve batches of Relinqing^®^ samples were pulverized and screened through 250 μm sieves. The obtained fine powder (granules 1.00 g, capsule 0.30 g and tablet 0.50 g, equivalent to 3.33 g of the original medicine, respectively) was accurately weighed and placed in a 100 mL volumetric conical flask with stopper after adding 50 mL of 50% (v/v) methanol to the conical flask, the mixture was extracted with the aid of ultrasound (250 W, 40 kHz) for 60 min according to the Chinese Pharmacopeia (2010 Edition) [25]. A certain amount of 50% (v/v) methanol was then added to the mixture until the original weight was achieved. An appropriate amount of extracting solution was diluted 10-fold. The final solution was filtered through a syringe filter (0.22 μm). The filtrate was stored as sample solution until analysis.

### 3.3. Preparation of Standard Solutions

The standard stock solutions of: (1) gallic acid; (2) protocatechuic acid; (3) vanillic acid; (4) syringic acid; (5) catechin; (6) schizandriside; (7) quercitrin; (8) quercetin; (9) kaempferol, *p*-methoxybenzoic acid (internal standard 1) and naringin (internal standard 2) were prepared in 50% (v/v) methanol to the final concentrations of 1 mg·mL^−1^. Then these analytes were appropriately diluted with 50% (v/v) methanol to provide at nine different concentrations from 10 μg·mL^−1^ to 1 ng·mL^−1^, which were used to establish the calibration curves.

### 3.4. Chromatographic and Mass Spectrometric Conditions

RRLC was performed on an Agilent 1200 system equipped with an Agilent 6410 Triple Quadruple with an electrospray ion source (ESI). A gradient elution system consisting of (A) water and (B) acetonitrile was used. The gradient was started at 5% B and maintained for 2 min at a flow rate of 0.3 mL·min^−1^, followed by a linear increase to 90% B at the same flow rate from 2 min to 8 min, then maintained for 2 min at 90% B. Chromatographic separation was performed on an Agilent XDB C_18_ column (2.1 mm × 100 mm, 3.5 μm). The mass spectrometer was operated in the negative multiple reaction monitoring (MRM) mode. The MS analysis conditions were set as follows: nitrogen used as a drying gas at a flow rate of 10 L·min^−1^ with a temperature of 35 °C, a nebulizer pressure of 30 psi, and an electrospray capillary voltage of 4,000 V. High-purity helium was used as the collision gas.

## 4. Conclusions

In this paper, the RRLC method coupled with triple quadrupole mass spectrometry was proven to be a reliable and powerful technique for simultaneous determination of nine active components, including four phenolic acids, four flavonoids and a lignin, in three dosage forms of Relinqing^®^. The MRM mode of QQQ MS enabled identification of the target compounds with high selectivity even at very low concentration by comparison with standards. The rapid analysis performed facilitated the efficient identification and quantification of the target components in Relinqing^®^. The developed method was demonstrated to be simple, sensitive and efficient, which is significant and comprehensive for quality control of Relinqing^®^. The established methodology may apply to or widely adapted to other sources to speed up the internationalization of Chinese medicine.

## References

[1] Li S.P., Zhao J., Yang B. (2011). Strategies for quality control of Chinese medicines. J. Pharm. Biomed. Anal..

[2] Liang X.M., Jin Y., Wang Y.P., Jin G.W., Fu Q., Xiao Y.S. (2009). Qualitative and quantitative analysis in quality control of traditional Chinese medicines. J. Chromatogr. A.

[3] Shekarchi M., Hajimehdipoor H., Saeidnia S., Gohari A.R. (2012). Comparative study of rosmarinic acid content in some plants of Labiatae family. Pharmacogn. Mag..

[4] Jiang Y., David B., Tu P.F., Barbin Y. (2010). Recent analytical approaches in quality control of traditional Chinese medicines—A review. Anal. Chim. Acta.

[5] Liang X., Zhang L., Zhang X., Dai W.X., Li H.Y., Hu L.W., Liu H., Su J., Zhang W.D. (2010). Qualitative and quantitative analysis of traditional Chinese medicine Niu Huang Jie Du Pill using ultra performance liquid chromatography coupled with tunable UV detector and rapid resolution liquid chromatography coupled with time-of-flight tandem mass spectrometry. J. Pharm. Biomed. Anal..

[6] Hu F.L., Deng C.H., Liu Y., Zhang X.M. (2009). Quantitative determination of chlorogenic acid in Honeysuckle using microwave-assisted extraction followed by nano-LC-ESI mass spectrometry. Talanta.

[7] Song L.R., Ding X.L., Zang Z.Y., Hong X. (2001). Dictionary of Modern TCD.

[8] Jiangsu New Medicinal College (1977). Dictionary of Chinese Herbal Medicine.

[9] Ren G.Y., Chang F.G., Lu S.L., Zhong H.L., Zhang G.L. (1995). Pharmacological studies of *Polygonum capitatum* Buch-Ham ex D Don. Zhongguo Zhong Yao Za Zhi.

[10] Yan X.L., Li C.Q., Liu Y.X., Chang X., Kang W.Y. (2010). Antioxidant activity of *Polygonum capitatum*. China Pharmacy.

[11] Li Y.M., Gong Y. (2007). The research progress on the chemical component and the pharmacology of *Polygotum capitatum* Ham ex D. Don. J. Guizhou Univ. (Nat. Sci.).

[12] Liu Z.J., Qi J., Zhu D.N., Yu B.Y. (2008). Chemical constituents from *Polygonum capitatum* and their antioxidation activities *in vitro*. Zhong Yao Cai.

[13] Yang Y., Cai F., Yang Q., Yang Y.B., Sun L.N., Chen W.S. (2009). Study on chemical constituents of *Polygonum capitatum* Buch.-Ham. ex D. Don(I). Acad. J. Second Military Med. Univ..

[14] Zhao H.X., Bai H., Li W., Wang Y.S., Liu Y.J., Liu A.Q. (2011). Chemical constituents from *Polygonum capitatum*. Nat. Prod. Res. Dev..

[15] Yu M., Li Z.L., Li N., Li X. (2008). Chemical constituents of the aerial parts of *Polygonum capitatum*. J. Shenyang Pharmaceut. Univ..

[16] Liao S.G., Zhang L.J., Sun F., Zhang J.J., Chen A.Y., Lan Y.Y., Li Y.J., Wang A.M., He X., Xiong Y. (2011). Antibacterial and anti-inflammatory effects of extracts and fractions from *Polygonum capitatum*. J. Ethnopharmacol..

[17] Watson D.G., Oliveira E.J. (1999). Solid-phase extraction and gas chromatography—Mass spectrometry determination of kaempferol and quercetin in human urine after consumption of Ginkgo biloba tablets. J. Chromatogr. B.

[18] Boue S.M., Carter-Weintjies C.H., Shih B.Y., Cleveland T.E. (2003). Identification of flavone aglycones and glycosides in soybean pods by liquid chromatography—Tandem mass spectrometry. J. Chromatogr. A.

[19] Zhao H.X., Bai H., Li W., Wang Y.S. (2010). Study on lignans of *Polygonum capitatum*. Zhong Yao Cai.

[20] Li Y.S., Wang X.P., Wan D.G., Wu H.M. (2011). The thinking of study and development of series of Huoxiang Zhengqi preparations. Pharmacy Clin. Chin. Materia Medica.

[21] Xie Y., Zhang L.Y., Liang B., Li M.L., Tang J.W. (2009). Determination of quercitrin in *Polygonum capitatum* and Relinqing granules by HPLC. China J. Chin. Materia Medica.

[22] Yang B.B., Feng R., Wang W.C., Zhang L.Y., Ye X.M., Wang Y., Wang M.Z. (2008). Quantitative analysis of three active constituents in Miao regional herb *Polygonum capitatum* by HPLC/DAD/MS. Chin. J. Pharm. Anal..

[23] Steinmann D., Ganzera M. (2011). Recent advances on HPLC/MS in medicinal plant analysis. J. Pharm. Biomed. Anal..

[24] Zhang Y., Xu H., Chen X., Chen C., Wang H., Meng F., Yang H., Huang L. (2011). Simultaneous quantification of 17 constituents from Yuanhu Zhitong tablet using rapid resolution liquid chromatography coupled with a triple quadrupole electrospray tandem mass spectrometry. J. Pharm. Biomed. Anal..

[25] Peng J.B., Jia H.M., Liu Y.T., Zhang H.W., Dong S., Zou Z.M. (2011). Qualitative and quantitative characterization of chemical constituents in Xin-Ke-Shu preparations by liquid chromatography coupled with a LTQ Orbitrap mass spectrometer. J. Pharm. Biomed. Anal..

